# Visceral leishmaniasis associated with macrophage activation syndrome: Case report and literature review

**DOI:** 10.1016/j.idcr.2021.e01247

**Published:** 2021-08-10

**Authors:** Boutaina Mouhoub, Mohammed Bensalah, Abdelilah Berhili, Ali Azghar, Jalila El Malki, Imane El Mezgueldi, Oumaima Nassiri, Mounia Slaoui, Achraf Miri, Chaymae Rochdi, Noufissa Benajiba, Rachid Seddik

**Affiliations:** a Central Laboratory, University Hospital Center Mohamed VI Oujda, Morocco; b Faculty of Medicine and Pharmacy Oujda, Mohammed First University Oujda, Morocco; c Pediatric Service, University Hospital Center Mohamed VI Oujda, Morocco

**Keywords:** VL, visceral leishmaniasis, MAS, macrophage activation syndrome, LDH, lactate dehydrogenase, MGV, mean globular volume, MCH, mean corpuscular hemoglobin, PT, prothrombin time, DIC, disseminated intravascular coagulation, HLH, Hemophagocytic Lymphohistiocytosis, Visceral leishmaniasis, Macrophage activation syndrome, Sand flies, Protozoa parasite, Morocco

## Abstract

**Background:**

The combination of visceral leishmaniasis (VL) and macrophage activation syndrome (MAS) makes the diagnosis difficult due to their similar clinical presentation, with a poor prognosis especially since the treatment is still poorly codified.

We report the case of a 17-month-old female patient from Berkane, presenting for a 3 months history of anarchic fever with anemic syndrome made up of pallor and hemorrhagic syndrome made up of epistaxis. Physical examination revealed a temperature of 39 ° C, lower limbsedema, paleness of skin and mucous membranes, gingival petechiae, bleached hair, and hepatosplenomegaly.

**Case presentation:**

The complete blood count showed pancytopenia with deep aregenerative normochromic normocytic anemia at 3 g/dL, leukocytes were at 4860/mm 3 with neutropenia at 680/mm 3 and thrombocytopenia at 12.000/mm^3^, the blood smear was without abnormality. These anomalies were associated with a hypoalbunemia, hypertriglyceridemia, hyperferritinemia, lactate dehydrogenase (LDH) level was at 337 IU/L, low prothrombin time (PT) at 56 % and fibrinogen level at 1 g/L. The direct Coombs test was positive. Examination of the myelogram revealed the presence of leishmania bodies and figures of hemophagocytosis. A diagnosis of visceral leishmaniasis associated with MAS was made.

The patient was put on liposomal amphotericin B and corticosteroid therapy with good clinical and biological evolution and good therapeutic tolerance.

**Conclusion:**

The association of VL and MAS remains rare and should be evoked even in non-endemic areas since late diagnosis worsens the prognosis and may even be responsible for the death of patients despite an aggressive treatment.

## Introduction

Visceral leishmaniasis is a parasitic zoonosis caused by a flagellated parasite of the genus Leishmania, transmitted by bites of female sandflies. Morocco is an endemic country and leishmaniasis represents a real public health problem in this country [1].

Macrophage activation syndrome (MAS) is an anatomo-clinical entity due to inappropriate activation and proliferation of the lymphohistiocytic lineage [2,3].

Indeed, the combination of these two clinical entities makes diagnosis difficult due to their similar clinical presentation [4].

Thus, the prognosis is worse especially since the treatment is still poorly codified [2].

We report a new observation about the combination of these two entities in a 17 months old infant.

## Observation

We report the case of a 17-month-old female patient from Berkane, born from a consanguineous marriage with the notion of the death of a sister at the age of 6 (unknownetiology), with no other pathological history. She presented for an anarchic fever evolving for 3 months with the anemic syndrome and hemorrhagic syndrome made up of epistaxis with stature retardation. Physical examination revealed a temperature of 39 ° C, lower limbsedema, paleness of skin and mucous membranes, gingival petechiae, bleached hair and hepatosplenomegaly. Other exams are unremarkable.

**Fig. 1:**
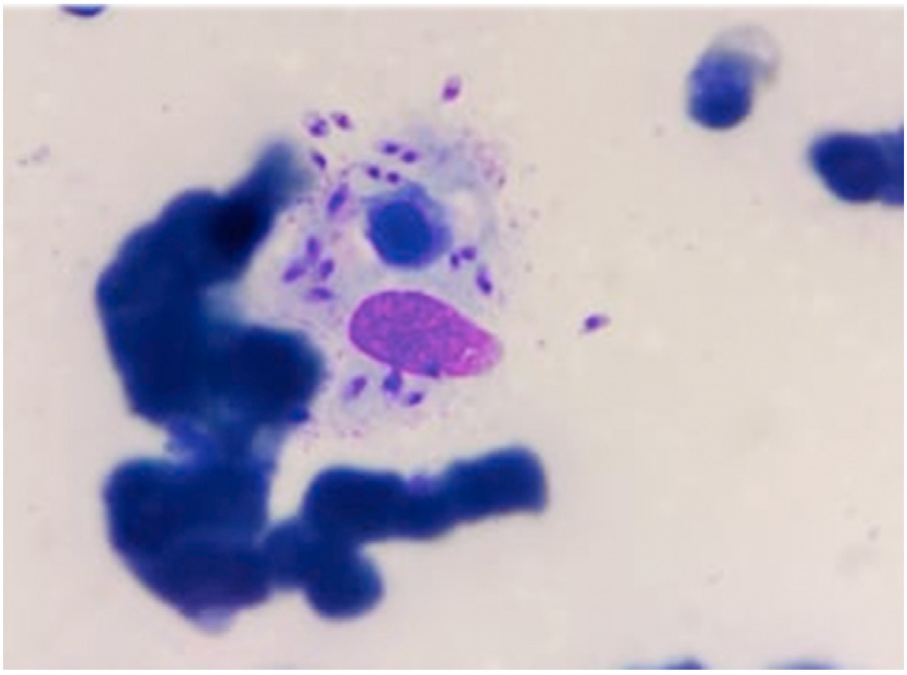
Hemophagocytosis with intramacrophagic Leishmania bodies.

**Fig. 2:**
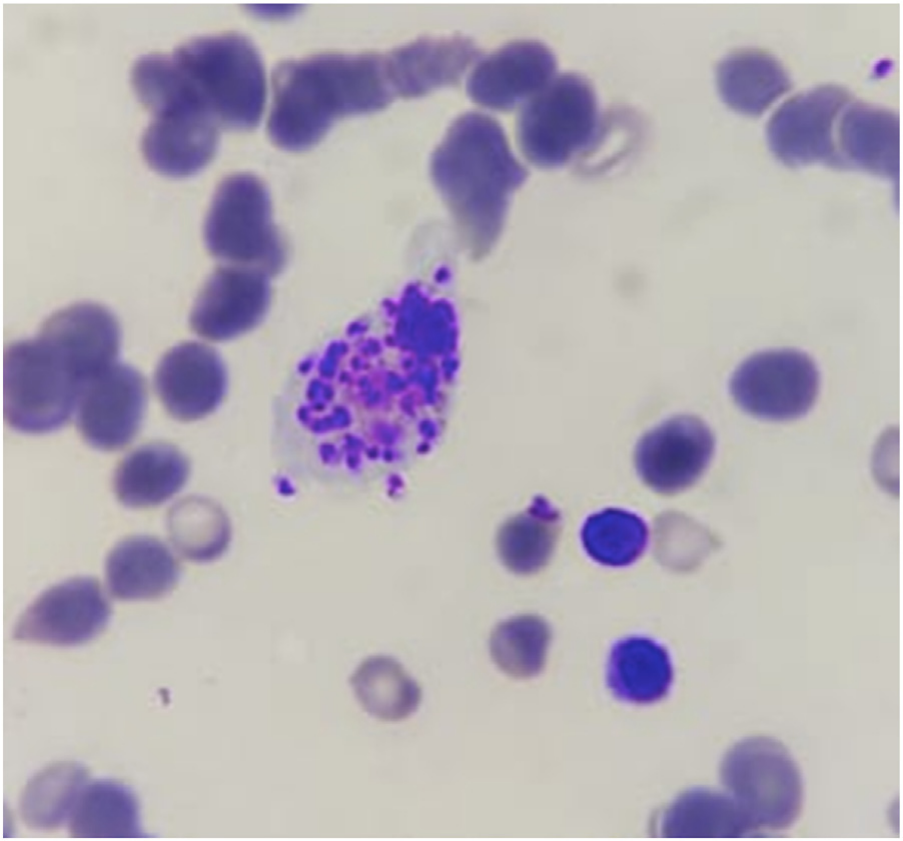
Bodies of intramacrophagic Leishmanias.

The complete blood count showed pancytopenia made up of deep aregenerative normochromic normocyticanemia at 3 g/dL, with reticulocyte count at 76.700/mm^3^, leukocytes at 4860/mm^3^ made up of neutropenia at 680/mm^3^ and thrombocytopenia at 12.000/mm^3^. The blood smear showed no abnormality. We also observed a hypoalbuminemia at 13 g/L, hypertriglyceridemia at 4.57 g/L, hyperferritinemia at 626ug/L, lactate dehydrogenase (LDH) level at 337IU/L, low prothrombin time (PT) at 56 %, and fibrinogen level at 1 g/L. The direct Coombs test was also positive. The hepatic assessment and the renal function are without abnormality. (Table 1) in front of these clinical and biological features, the clinicians taking charge of the patient evoked the diagnosis of MAS. Thus, a cytological study of the bone marrow in search of the features of hemophagocytosis was necessary to confirm the diagnosis. Careful examination of the myelogram revealed the presence of leishmania bodies with figures of hemophagocytosis (Fig. 1, Fig. 2). The diagnosis of visceral leishmaniasis associated with a MAS was retained.

**Table 1:** Laboratory results.

Biological Parameters	Case	Reference range
WHITE BLOOD CELLS	4860 /μL	4 000,00–10.000/μL
HEMOGLOBIN	3.6 g/dL	9,5−14 g/dL
MGV	9350 fL	68−86 fL
MCH	2610 pg	23−31 pg
PLATELET RATES	12,000 /μL	150000−550000/μL
PNNEUTROPHILS	680 /μL	1000−8500/μL
LYMPHOCYTES	3850 /μL	3000−13000/μL
MONOCYTES	330 /μL	200−1000/μL
RETICULOCYTES	76.700/μL	120.000/μL
Triglyceridemia	4.57 g/L	<1,5 g/L
Albuminemia	13 g/L	38−54 g/L
Ferritinemia	626 ug/L	15−120 ug/L
LDH	337IU/L	125−243 UI/L
Viral serology	Negative	_
PT	56 %	70−100%
Fibrinogen	1 g/L	2−4 g/L

The patient was put on liposomal amphotericin B and corticosteroid therapy with good clinical and biological progress and good therapeutic tolerance.

## Discussion

Leishmaniasis is a parasitic infection caused by flagellated protozoa of the genus Leishmania. Transmission is caused by a vector: female sand flies following a bite during a blood meal. It is the most common mode of Leishman's infection. Although rare, other modes of transmission have been described: accidental ingestion, crushing infected sandflies on damaged skin, and contact with infected blood (transfusion and needle exchange) [1].

There are two clinical forms of leishmaniasis in Morocco: cutaneous leishmaniasis distributed in the center of the country, the South and the South-East of the Atlas chain, and visceral leishmaniasis observed in the North, mainly in the regions of Nador, al- Hossima, Tetouan, Taza, Taounate, Sidi-Kacem, Meknes, and Fès [1,5]. This last form, the most serious, is due to Leishmania infantum, the reservoir of which is the dog. However, humans represent only a parasitic dead-end and are only infected accidentally. It is usually seen in children (the average age is between 3.5 and 4 years), this can be explained by the immaturity of their immune system. The usual clinical picture is made up of the well-known triad: chaotic fever, pallor, and splenomegaly [1,5,6].

Macrophage activation syndrome (MAS), also known as lymphohistiocytic hemophagocytosis, is a clinical biological entity caused by uncontrolled activation and proliferation of cells of the lymphohistiocytic lineage responsible for the destruction of multiple organs [2,4,7]. None of the clinical symptoms or laboratory abnormalities are specific to MAS. Indeed, the clinical presentation of MAS is often brutal with multiple organ involvement which is sometimes misleading. Clinical symptoms are variably made of a deterioration of the general status, a high fever, an organomegaly, dermatological manifestations (morbilliform, rash or purpura), and neurological manifestations (convulsions, signs of localization). Respiratory and digestive signs are rarer [2]. Laboratory abnormalities during MAS are numerous and non-specific. The blood count most often shows a bi or pancytopenia: thrombocytopenia (central or peripheral related to disseminated intravascular coagulation (DIC), non-regenerative normochromic normocytic anemia, while leukopenia is less frequent and of late-onset. Beside, hemostatic disturbances may also be present, including hypofibrinogenemia, isolated or associated with a prolonged PT. The latter can be due to a simple activation of coagulation, or even to a real DIC. On the biochemical level, LDH is constantly increased. Thus, there is often anearly hypertriglyceridemia, which can reach more than 10 times its normal value, associated with a significant increase in ferritinemia following cell lysis by macrophage activation. Biological liver damage is found in 40 % of cases [8]. Therefore, it is the association of clinical and biological signs (Table 2) that is very evocative and that leads to a strong presumption [9]. In addition, histological evidence of hemophagocytosis images is of great diagnostic value and remains the gold standard, although it is often absent at the very beginning, which can complicate the diagnosis and worsen the prognosis [10].

**Table 2:** Diagnostic criteria for macrophage activation syndrome [9].

Fever
Splenomegaly
Cytopenias of more than two lineages
Blood triglyceride elevation and / or fibrinogen decrease
Hemophagocytosis in the marrow, liver, or lymph node
Low NK activity
Elevation of serum ferritin above 500 μg/L
Soluble IL-2 receptor increased above 2400 UI/mL.
Diagnosis of SAM on more than six criteria

MAS can be primary, or secondary to various infectious, hematological or autoimmune conditions. The etiological diagnosis, therefore requires an exhaustive assessment which often remains non-contributory. The clinical presentation is usually dominated by manifestations secondary to MAS, thus masking specific signs of the causative pathology. Likewise, MAS can be masked by the manifestations of the underlying pathologies that trigger it [2].

Infections are responsible for around 50 % of MAS cases, mainly viral infections (CMV, EBV, HSV) then mycobacteria (tuberculosis), intracellular bacteria, pyrogens, and finally parasites [2]. Among the parasitic causes, we find visceral leishmaniasis, the association of which with SAM presents a diagnostic challenge due to the rarity and similarity of clinical and biological features which can lead to diagnostic and therapeutic delay. In fact, 13 collected cases of VL out of 276 were associated with MAS, equivalent to a prevalence of 4.7% as demonstrated in a study carried out in Algeria over 10 years [11]. Moreover, our observation illustrates the diagnostic difficulties encountered in the presence of MAS, especially since our patient comes from a non-endemic region, and thus the probability of visceral leishmaniasis is low.

According to the HLH-2004 (Hemophagocytic Lymphohistiocytosis) criteria [9], the diagnosis of MAS was retained in our patient on the presence of fever, splenomegaly, pancytopenia, hypertriglyceridemia, hyperferritinemia, hypofibrinogenemia, and on the presence of figures of hemophagocytosison the myelogram. For the leishmania bodies, their discovery was fortuitous in the marrow, which made it possible to establish the diagnosis early without other complementary investigations and thus enabled a quickly established specific treatment of MAS in addition to the anti-infectious treatment. Indeed, in a series of 56 cases of MAS-LV association, the first marrow failed to reveal Leishmania bodies in 64.7 % of cases and figures of hemophagocytosis in 36.3 % of cases [4].

The direct Coombs test was positive in our patient. This finding has also been reported by Higel et al. and Tazi et al. in two infants of 20 months and 7 months with the same clinical presentation as for our case. The latter, of unexplained pathogenicity, may be due to polyclonal lymphocytic hyperreactivity [4,12].

Consequently, and fortunately for our patient, the diagnosis of visceral leishmaniasis associated with MAS was made 3 days after his hospitalization and after performing a single marrow without the need of serology or other additional investigations.

The standard treatment for visceral leishmaniasis is liposomal amphotericin B, which is effectivein more than 90 % of cases, with a short treatment duration and good tolerance. The other treatment used is meglumine antimoniate with a longer treatment duration for at least 20 consecutive days and the treatment should be continued until the parasites disappear. In the event of recurrence, the treatment must be immediately restarted with the same daily doses [2,11]. Treatment of secondary MAS is not codified. Recent advances have made it possible to better understand its pathophysiology and to adapt treatments. It is based on etiological treatment, that can be depending on cases, associated with intravenous immunoglobulins, short-course steroids or immunosuppressants [2,11]. In our case, the patient received liposomal amphotericin B combined with corticosteroid therapy. The outcome was clinically and biologically favorable with good therapeutic tolerance.

The early diagnosis of the association of VL and MAS in our case made it possible to initiate specific and anti-infectious treatment early and therefore improve the prognosis. The latter becomes worse when there is a delay in the diagnosis and death could occur despite aggressive treatment and close monitoring [2,4,11].

## Conclusion

SAM is a serious entity with multiple etiologies, infections ones being at the top of the list. A rapid exhaustive infectious assessment is necessary. The association of VL and MAS remains rare and should be sought even in non-endemic areas because late diagnosis makes the prognosis worse and may even be responsible for the death of patients despite aggressive treatment and close monitoring.

## Declaration of Competing Interest

The authors declare that they have no conflict of interest.

## Funding

There is no special funding for this study.

## Authors contributions

**MOUHOUB Boutaina:** Make diagnosis, contribute to wrote the manuscript and performed experiments.

**BENSALAH Mohammed:** analyzed the data.

**Abdelilah BERHILI:** conceived and designed the experiments.

**Ali AZGHAR:** performed experiments.

**Jalila EL MALKI:** performed experiments.

**Imane EL MEZGUELDI:** performed experiments.

**Oumaima NASSIRI:** performed experiments.

**Mounia SLAOUI:** performed experiments.

**Achraf MIRI:** traduction, write and approved the final manuscript.

**Chaymae ROCHDI**: write and approved the final manuscript

**Noufissa BENAJIBA:** performed experiments.

**Rachid SEDDIK:** supervise and direct the study

## Consent

Written informed consent was obtained from the patient for publication of this case report and accompanying images.
